# X-FASNet: cross-scale feature-aware with self-attention network for cognitive decline assessment in Alzheimer's disease

**DOI:** 10.3389/fneur.2025.1630838

**Published:** 2025-08-12

**Authors:** Wenhui Chen, Shunwu Xu, Yiran Peng, Hong Zhang, Jian Zhang, Huaihao Zheng, Hao Yan, Zhaowen Chen

**Affiliations:** ^1^Key Laboratory of Nondestructive Testing, Fujian Polytechnic Normal University, Fuzhou, China; ^2^Faculty of Innovation Engineering, Macau University of Science and Technology, Avenida Wai Long, Macau, China

**Keywords:** Alzheimer's disease, multi-scale model, cross-scale feature-aware self-attention, feature fusion, cognitive decline assessment

## Abstract

Early diagnosis of Alzheimer's disease is critical for effective therapeutic intervention. The progressive nature of cognitive decline requires precise computational methods to detect subtle neuroanatomical changes in prodromal stages. Current multi-scale neural networks have limited cross-scale feature integration capabilities, which constrain their effectiveness in identifying early neurodegenerative markers. This paper presents an Efficient Cross-Scale Feature-Aware Self-Attention Network (X-FASNet) designed to address these limitations through systematic hierarchical representation learning. The proposed architecture implements a dual-pathway multi-scale feature extraction approach to identify discriminative neuroanatomical patterns across various spatial resolutions, while integrating a novel cross-scale feature-aware self-attention module that enhances inter-scale information exchange and captures long-range dependencies. Quantitative evaluations on the DPC-SF dataset demonstrate that X-FASNet achieves superior performance with 93.7% accuracy and 0.973 F1-score, outperforming CONVADD by 10.8 percentage points in accuracy and 0.118 in F1-score, while also surpassing EfficientB2 on key performance metrics. Comprehensive experimentation across multiple neuroimaging datasets confirms that X-FASNet provides an effective computational framework for neurodegeneration assessment, characterized by enhanced detection of subtle anatomical variations and improved pathological pattern recognition.

## 1 Introduction

Alzheimer's disease (AD), the most prevalent neurodegenerative disorder worldwide, is characterized by progressive deterioration of cognitive functions and memory systems (1). Clinically manifested through impaired executive function, visuospatial deficits, and eventual loss of autonomy, AD pathogenesis involves complex interactions between amyloid-beta deposition, tau pathology, and neuroinflammation (2). The disease trajectory typically progresses through three clinically defined stages: cognitively normal (CN), mild cognitive impairment (MCI), and full dementia (AD), with MCI representing a critical window for therapeutic intervention (3, 4).

The insidious nature of AD progression underscores the imperative for early detection methodologies. Current diagnostic paradigms, which rely on neuropsychological assessments (e.g., MMSE, ADAS-Cog), exhibit limited sensitivity to pre-clinical stages. At the same time, cerebrospinal fluid biomarkers remain invasive and cost-prohibitive for population screening (5). Neuroimaging modalities, particularly structural MRI, have emerged as crucial tools for identifying early patterns of neurodegeneration. Automated analysis of MRI-derived biomarkers, including hippocampal atrophy, cortical thinning, and white matter hyperintensities, provides quantifiable metrics that correlate with cognitive decline trajectories (6).

Deep learning architectures have demonstrated remarkable success in decoding complex neuroimaging signatures of Alzheimer's disease (AD) progression (7, 8). Convolutional neural networks (CNNs) excel at capturing hierarchical representations of neurodegeneration patterns through multi-scale feature learning. However, current implementations face three fundamental limitations: (1) inadequate integration of cross-scale contextual information, leading to suboptimal utilization of complementary features across spatial resolutions; (2) limited capacity to model long-range dependencies between distributed neural substrates affected in AD; (3) insufficient attention to neuroanatomically plausible regions of interest, compromising model interpretability (9).

Recent advances in multi-scale architectures and attention mechanisms offer promising solutions to these challenges. While residual connections and dense blocks enhance feature reuse (10), and self-attention modules improve global context modeling (11), existing implementations often neglect the hierarchical nature of neurodegenerative processes. The progressive spatial expansion of AD pathology—from medial temporal lobe structures to association cortices—demands architectures capable of adaptively weighting local and global neurodegeneration signatures (12).

Despite their promising capabilities, current multi-scale convolutional neural networks face two critical limitations (13, 14). First, these architectures struggle to effectively integrate information across different spatial scales during feature fusion processes. This integration challenge frequently results in feature redundancy or information loss, ultimately compromising classification performance. Second, conventional convolutional operations are fundamentally constrained by their local receptive fields, preventing the capture of long-range dependencies between anatomically distant but functionally related regions—a limitation that significantly reduces feature representation capacity.

To address these challenges, we propose the Cross-Scale Feature-Aware Self-Attention Network (X-FASNet). Our architecture implements two complementary innovations: (1) a multi-scale feature extraction framework specifically designed to capture comprehensive neuroanatomical information from Alzheimer's neuroimaging data, and (2) a cross-scale feature-aware self-attention mechanism that facilitates effective information fusion across different scales. This integrated approach optimizes cross-scale feature interactions while simultaneously modeling long-range dependencies, substantially enhancing the network's representational capabilities and diagnostic accuracy.

The main contributions of this paper are summarized as follows:

- We design a dual-pathway multi-scale feature extraction module by employing parallel 5 × 5 and 7 × 7 convolutional pathways with subsequent depth-separable convolutions. Our approach extracts both fine-grained local features critical for subtle pathological changes and broader contextual patterns that characterize disease progression. Ablation studies demonstrate this dual-pathway architecture improves classification accuracy by 4.7% compared to single-scale approaches.
- We introduce a cross-scale feature-aware self-attention mechanism that enables dynamic feature integration across spatial scales while modeling long-range dependencies between anatomically distant brain regions affected by AD pathology. This mechanism enhances representation capacity as evidenced by a 6.5% improvement in multi-class classification accuracy over conventional feature fusion methods, with visualization analyses confirming focus on neuroanatomically relevant regions.
- We propose X-FASNet, an integrated architecture that combines these contributions to address the limitations of conventional multi-scale networks for Alzheimer's disease diagnosis. Extensive evaluations demonstrate state-of-the-art performance across multiple datasets: on DPC-SF, X-FASNet achieves 93.7% accuracy and 0.973 F1-score, outperforming CONVADD (15) by 10.8% in accuracy; on DPC-Pre, it attains 95.5% accuracy with an AUC of 0.997, exceeding current leading models while maintaining clinically relevant interpretability.

## 2 Related work

### 2.1 Machine learning based diagnostics for Alzheimer's detection

Considering that traditional diagnostic methods cannot meet the demand for early detection of Alzheimer's disease. In recent years, machine learning has garnered significant attention in Alzheimer's disease (AD) diagnosis. First, feature mining of medical images is performed by locating key regions through feature extraction algorithms, and then, based on the classification model, features are recognized. Compared to traditional diagnostic methods, machine learning based diagnostic methods are relatively objective and more suitable for large-scale screening. Change et al. (9) summarized several novel biomarkers and, using machine learning algorithms and multivariate analysis, distinguished between patients with AD and healthy individuals. Experiments have demonstrated that combining machine learning algorithms with multivariate analysis can improve the accuracy of Alzheimer's disease (AD) diagnosis. Fan et al. (16) employed a support vector machine (SVM) model to classify and predict the various stages of Alzheimer's disease, utilizing MRI imaging data to facilitate an efficient diagnosis of the disease. Alghamedy et al. (17) proposed a multi-model approach based on machine learning for medical imaging studies, aiming to classify and detect Alzheimer's disease. Neffati et al. (18) proposed a method based on downsized kernel principal component analysis (DKPCA) and multi-class support vector machines for classifying MRI images in different states of Alzheimer's disease. Beheshti et al. (6) developed a feature selection method based on whole-brain voxel analysis of MRI data for the computer-aided diagnosis (CAD) framework. Dong et al. (19) proposed a machine learning method based on functional connectivity of whole-brain connectome for probing the network substrate of cognitive deficits in Alzheimer's disease. Alatrany et al. (20) proposed a machine learning model with interpretability oriented toward early diagnosis of Alzheimer's disease (AD). Specifically, the method introduces Class Association Rules (CAR) and Stable and Interpretable Rule Set for classification (SIRUS) model to enhance the interpretability of the method. Although machine learning based methods have demonstrated better performance in early diagnosis, they usually rely on manually extracted features, which tend to lead to an insufficient generalization ability of the model when facing physiological differences among individual patients.

### 2.2 Deep learning based diagnostics for Alzheimer's detection

In recent years, deep learning methods have become a significant focus of research in the field of early Alzheimer's disease diagnosis. Using deep learning methods, researchers can automatically extract deep features from various types of medical images to enhance the accuracy and robustness of diagnoses (21). AbdulAzeem et al. (22) proposed a convolutional neural network-based end-to-end image classification framework for Alzheimer's disease, achieving promising results. Al Shehri (23) proposed a deep learning based solution to diagnose and classify Alzheimer's disease by using the CNN architecture of DenseNet-169 and ResNet-50. Yang et al. (12) evaluated the performance of deep learning algorithms in differentiating between patients diagnosed with Alzheimer's disease using baseline MRI brain data. They also combined the features extracted from the neural network with other baseline biomarkers to create prognostic markers for Alzheimer's disease. Liang et al. (24) proposed a distillation multi-residual network (DMRNet) with dilated classifiers and self-distillation for the early diagnosis of Alzheimer's disease (AD), aiming to explore the hidden knowledge between each feature. Khan et al. (25) proposed a deep learning-based multi-class classification method to distinguish the stages of early Alzheimer's disease diagnosis. Through extensive experimental validation, it is shown that the proposed method has good classification performance. Wu et al. (26) proposed a three-dimensional (3D) transfer learning network based on two-dimensional (2D) transfer learning to classify Alzheimer's disease (AD) and normal groups from MRI images. Jiang et al. (27) proposed a new method for classifying images of Alzheimer's disease based on the external-attention mechanism. This approach utilizes the external-attention mechanism to classify images of Alzheimer's disease. To further improve representational capacity, several studies have explored multi-scale feature extraction strategies. Song et al. (28) proposed a three-dimensional multi-scale CNN model for feature extraction, which achieves better overall performance. Tu et al. (29) proposed a multi-modal feature transformation approach for AD diagnosis, which extracts complementary features from multiple modalities to enhance the representation of Alzheimer's-related patterns. Although these approaches attempt to extract multi-scale information, they still rely heavily on convolutional architectures, which are limited in modeling long-range spatial dependencies. In summary, although existing deep learning methods have achieved encouraging progress in the early diagnosis of Alzheimer's disease, many of these methods primarily focus on either local or short-range features. Consequently, there are still deficiencies in multi-scale feature modeling and long-range dependency capture, which limit the ability to extract highly discriminative information of Alzheimer's disease.

## 3 X-FASNet for Alzheimer's disease detection

### 3.1 Method overview

**Figure 1:**
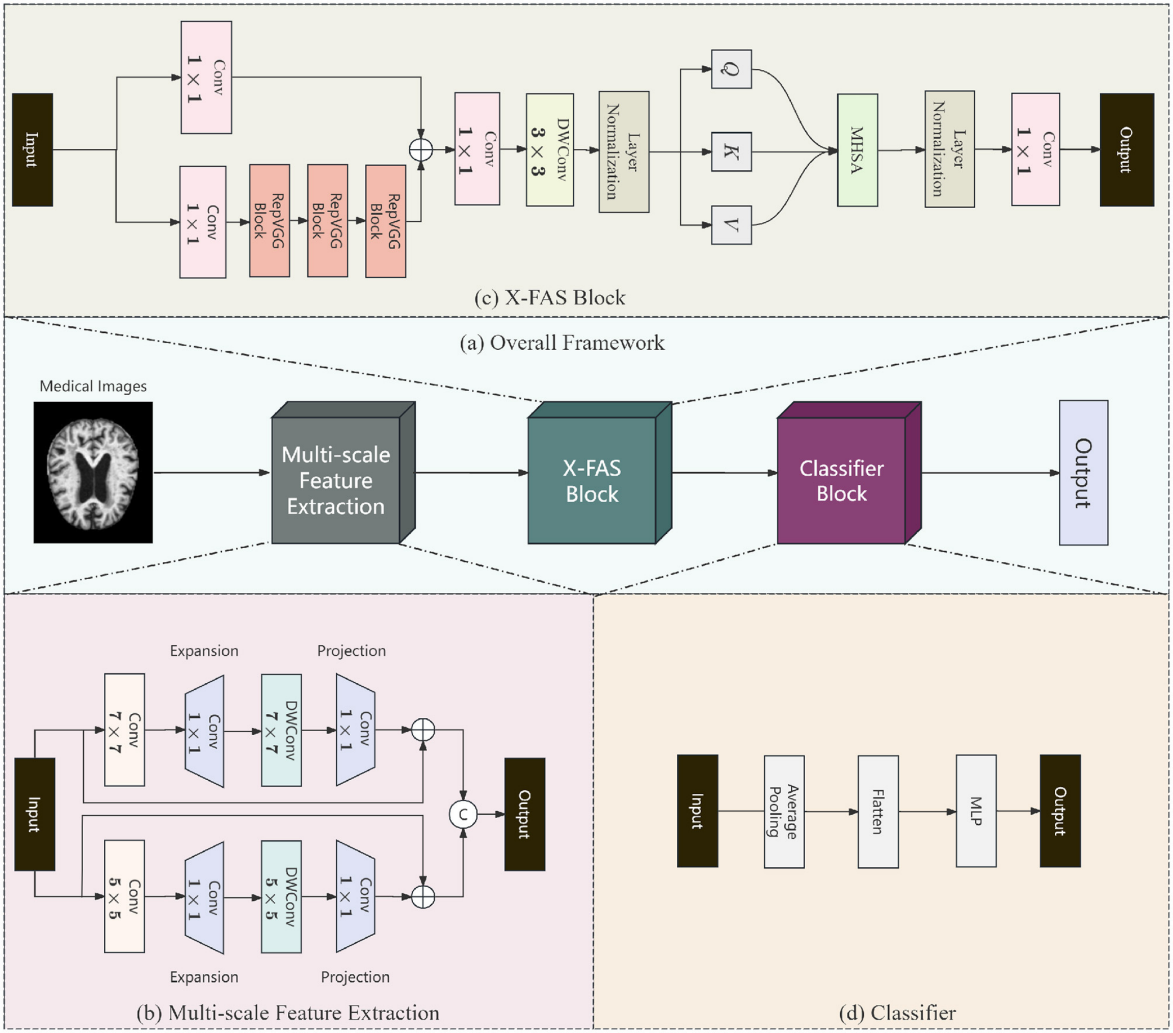
The overall model structure of X-FASNet. **(a)** Overall Framework; **(b)** Multi-scale Feature Extraction; **(c)** Cross-scale Feature-aware Self-attention (X-FAS) Block; **(d)** Classifier.

Although the Alzheimer's disease state recognition model based on multi-scale convolutional neural networks has made significant progress, it utilizes different sizes of convolutional kernels to extract various parts of the image, effectively capturing both global and local feature information. However, the process of fusing different-scale features is often accompanied by feature information imbalance, which makes the recognition model suffer from poor recognition performance. Furthermore, the absence of long-range dependencies means that the fused features often fail to capture the complex associations in the brain, which in turn affects the accurate diagnosis of early-stage conditions. For this reason, we propose an early recognition model for Alzheimer's disease based on X-FASNet. First, to capture key information at different scales, we constructed multi-scale features of the image using convolution with various kernel sizes. Second, to efficiently fuse multi-scale features, we propose the Cross-Scale Feature-Aware Self-Attention. On the one hand, multiscale features are merged through cross-scale feature fusion, and on the other hand, the long-range dependencies of the fused features are extracted using the self-attention mechanism. Finally, the extracted features are fed into the convolutional and pooling layers to obtain the final classification results. The structure of the overall model is shown in Figure 1.

### 3.2 Multi-scale feature extraction

To extract suitable features from Alzheimer's images more efficiently, we employ a multi-scale feature extraction strategy based on a two-way network. First, we target the image for shallow feature extraction using both large and small kernel convolutions. Among them, the use of large kernel convolution aims to extract global features and obtain more spatial information from the image. The small kernel convolution focuses more on the local detailed features of the image. Subsequently, we increased the number of channels by applying a 1 × 1 convolution, which enabled the better extraction of specificity information contained in the data during subsequent feature extraction. Then, to reduce the overall number of parameters in the network, we employ depth-separable convolution and shrink the number of channels through a 1 × 1 convolution to enhance the expressive ability of feature information. Not only that, for each way branch, we use residual concatenation to enhance the performance of the model. After obtaining the features of two-way branches, we combine these features to form multi-scale features. Let the alzheimer's image be *X*∈*R*^*H*×*W*×*C*^, and the following equation can be described as:


(1) $$\begin{array}{l} X^{1}=ReLU6\left(BN\left(Conv_{5\times 5}\left(X\right)\right)\right) \end{array}$$

(2) $$\begin{array}{l} X^{2}=ReLU6\left(BN\left(Conv_{7\times 7}\left(X\right)\right)\right) \end{array}$$

(3) $$\begin{array}{l} X^{3}=Conv_{1\times 1}\left(DWConv_{5\times 5}\left(Conv_{1\times 1}\left(X^{1}\right)\right)\right) \end{array}$$

(4) $$\begin{array}{l} X^{4}=Conv_{1\times 1}\left(DWConv_{7\times 7}\left(Conv_{1\times 1}\left(X^{2}\right)\right)\right) \end{array}$$

(5) $$\begin{array}{l} X^{Multi\text{-}scale}=Concat\left(X^{3},X^{4}\right) \end{array}$$

(6) $$\begin{array}{l} BN\left(x\right)=\alpha \cdot \frac{x-\mu_{B}}{\sqrt{\sigma_{B}^{2}+\epsilon }}+\beta \end{array}$$

(7) $$\begin{array}{l} ReLU6\left(x\right)=\min\left(\max\left(0,x\right),6\right) \end{array}$$

Where μ_*B*_ represents the mean of the batch, $\sigma_{B}^{2}$ represents the variance of the batch, and ε is a small positive number guaranteed to be computationally safe. α and β are a set of parameters that are constantly updated during network training. *Conv* represents the convolution operation, *BN* represents the batch normalization.

### 3.3 Cross-scale feature-aware self-attention

Multi-scale features extracted from medical images often contain rich local and global information. To enhance the characterization of features, we designed the Cross-Scale Feature-Aware Self-Attention module. The module is divided into two parts: cross-scale feature fusion and self-attention mechanism. Multi-scale features contain information at multiple levels, both locally and globally. To enhance the characterization ability of features at different scales, we introduce a cross-scale feature fusion module. The module adopts a two-branch structure. In one of the branches, we use a 1 × 1 convolution to adjust the number of channels, thereby realizing residual connectivity while maintaining the original information. In the other branch, the same 1 × 1 convolution is introduced to unify the channel dimensions, followed by three RepVGG blocks (30) for deep feature extraction. As a structural reparameterization unit, it can effectively improve the expression ability of features. Finally, the results of the two branches are summed element by element to realize the residual fusion of multi-scale features. This will provide stable and discriminative feature inputs for subsequent dependency mining. The process can be represented as:


(8) $$\begin{array}{l} X^{5}=Conv_{1\times 1}\left(X^{Multi\text{-}scale}\right) \end{array}$$

(9) $$\begin{array}{l} X^{6}=Conv_{1\times 1}\left(X^{Multi\text{-}scale}\right) \end{array}$$

(10) $$\begin{array}{l} X^{7}=RepVGG^{3}\left(X^{6}\right) \end{array}$$

(11) $$\begin{array}{l} RepVGG\left(X\right)=Conv_{3\times 3}\left(X\right)+Conv_{1\times 1}\left(X\right)+X \end{array}$$

(12) $$\begin{array}{l} X^{Cross\text{-}scale}=X^{6}+X^{7} \end{array}$$

Where *X*^*Cross*−*scale*^ is the feature after cross-scale fusion, *Conv* represents the convolution operation. *RepVGG*^3^ represents the operation of *RepVGG* Block performed three times.

Although cross-scale feature fusion efficiently integrates features at different scales, the dependencies between features are not fully utilized. For this reason, we introduce the multi-head self-attention mechanism. Compared to the traditional mechanism, the spatial dimensions of *K* and *V* are first reduced using a depth-separable convolution with a kernel size of *k*×*k* in *k* steps. Secondly, a bias term is added for learning (denoted as *B*). The process can be represented as:


(13) $$\begin{array}{l} X^{9}=DWConv_{3\times 3}\left(Conv_{1\times 1}\left(X^{Cross\text{-}scale}\right)\right) \end{array}$$

(14) $$\begin{array}{l} MHSA_{Q,K^{\prime },V^{\prime }}=Concat\left(head_{1},head_{2},\ldots ,head_{h\cdot W_{o}}\right) \end{array}$$

(15) $$\begin{array}{l} Q=LN\left(X^{9}\right)\times W_{Q} \end{array}$$

(16) $$\begin{array}{l} K^{\prime }=DWConv\left(LN\left(X^{9}\right)\right)\times W_{K} \end{array}$$

(17) $$\begin{array}{l} V^{\prime }=DWConv\left(LN\left(X^{9}\right)\right)\times W_{V} \end{array}$$

(18) $$\begin{array}{l} head_{h}=LSA\left(QW_{i}^{Q},K^{\prime }W_{i}^{K},V^{\prime }W_{i}^{V}\right) \end{array}$$

(19) $$\begin{array}{l} LSA\left(Q,K^{\prime },V^{\prime }\right)=Softmax\left(\frac{Q{K^{\prime }}^{T}}{\sqrt{d_{k}}}+B_{V}\right)V^{\prime } \end{array}$$

(20) $$\begin{array}{l} X^{Feature}=Conv_{1\times 1}\left(LN\left(MHSA\left(Q,K^{\prime },V^{\prime }\right)\right)\right) \end{array}$$

where *X*^*Feature*^ is the output feature, *d*_*k*_ is the dimension of the key vector *K*, *W*^*o*^, $W_{i}^{Q}$, $W_{i}^{K}$, and $W_{i}^{V}$ are all parameter matrices, and *LN* stands for layer normalization.

### 3.4 Classifier

After the high-level semantic feature extraction is completed, we design a structured classifier module for the final discrimination of Alzheimer's disease (AD). First, to reduce feature dimensionality, the input features are processed using average pooling. Second, the two-dimensional features are converted to one-dimensional using the Flatten operation. Finally, we utilize a compact multilayer perceptron to generate the prediction results through nonlinear mapping. The process can be represented as:


(21) $$\begin{array}{l} X^{10}=AvgPooling\left(X^{Feature}\right) \end{array}$$

(22) $$\begin{array}{l} O=MLP\left(Flatten\left(X^{10}\right)\right) \end{array}$$

Where *O* is the final output and *AvgPooling* is the average pooling operation.

## 4 Experiments

### 4.1 Dataset

Three public datasets are used to test our proposed model: the Kaggle Alzheimer's classification (KAC) dataset^1^ and the preliminary and semi-final rounds of the Disease Prediction Challenge of Alzheimer's Disease (DPC-Pre and DPC-SF).^2^ The KAC dataset comprises 6,400 MRI samples, categorized into four types of Alzheimer's disease images: Non-Demented, Very Mildly Demented, Mildly Demented, and Moderately Demented. DPC-Pre is a binary classification task that distinguishes between normal individuals (CN) and patients with Alzheimer's disease (AD), using 1,000 MRI images per class. DPC-SF extends the task to three classes—normal(CN), mild cognitive impairment (MCI), and AD, comprising 3,000, 3,000, and 4,000 samples, respectively. The detailed description of the three datasets is shown in Tables 1, 2.

**Table 1:** Sample distribution across different stages in the KAC dataset.

**Stage**	**Total samples**
Non-demented (ND)	2,400
Very-mild-demented (VMD)	2,240
Mild-demented (MD)	896
Moderate-demented (MoD)	64

**Table 2:** Sample distribution across different stages in the DPC-Pre and DPC-SF.

**Name**	**Stage**	**Total samples**
DPC-Pre	CN	1,000
	AD	1,000
DPC-SF	CN	3,000
	MCI	3,000
	AD	4,000

After image pre-processing, all images are resized to a fixed resolution of 168 × 168 pixels for model input. For the KAC dataset, we randomly split the data into 70% for training and 30% for evaluation. For the DPC datasets, both DPC-Pre and DPC-SF, 90% of the data are used to train the classification models, and the remaining 10% are reserved for evaluation.

### 4.2 Evaluation indicators

To comprehensively assess the performance of the classification models, we employ the following standard evaluation metrics: accuracy, precision, recall, *F*_1_-score, and the area under the ROC curve (AUC). These metrics are defined as:


(23) $$\begin{array}{l} Accuracy=\frac{TP+TN}{TP+TN+FP+FN} \end{array}$$

(24) $$\begin{array}{l} Precision=\frac{TP}{TP+FP} \end{array}$$

(25) $$\begin{array}{l} Recall=\frac{TP}{TP+FN} \end{array}$$

(26) $$\begin{array}{l} F_{1}=\frac{2\times Precision\times Recall}{Precision+Recall} \end{array}$$

Here, *TP*, *TN*, *FP*, and *FN* denote true positives, true negatives, false positives, and false negatives, respectively. AUC is the area under the Receiver Operating Characteristic (ROC) curve, which plots the *TP* rate against the false positive rate. A higher AUC value (closer to 1) indicates better classification performance.

### 4.3 Performance evaluation

To validate the effectiveness and generalization ability of our proposed model, we conduct comprehensive experiments on three Alzheimer's disease classification datasets: DPC-Pre, DPC-SF, and KAC. The proposed method is compared with a series of representative classifiers, including conventional deep convolution and attention-based models (10, 11, 30–39). The classification results of KAC are shown in Table 3. The classification results of DPC-Pre and DPC-SF are presented in Tables 4, 5, respectively.

**Table 3:** Performance comparison of different models in KAC.

**Name**	**Accuracy**	**Recall**	**Precision**	**F1-Score**	**AUC**
**Binary classification**					
ResNet18 (10)	0.7834	0.7952	0.7759	0.7854	0.8820
ResNet34 (10)	0.7771	0.7824	0.7639	0.7730	0.8783
DenseNet (32)	0.8122	0.8299	0.8128	0.8212	0.7452
SENet_18 (36)	0.7628	0.7673	0.7478	0.7574	0.8678
ECANet_18 (37)	0.7951	0.7799	0.7648	0.7723	0.8680
HRNet (39)	0.7628	0.7664	0.7543	0.7603	0.8499
GhostNet (35)	0.7538	0.7469	0.7311	0.7389	0.8393
RepVGG (30)	0.8104	0.8149	0.7950	0.8048	0.9158
Vision Transformer (11)	0.7340	0.6943	0.7034	0.6988	0.7465
CONVADD (15)	0.6827	0.6470	0.6527	0.6498	0.6848
Efficient B2 (41)	0.7333	0.6605	0.6846	0.6723	0.7452
**Our proposed**	**0.8431**	**0.8667**	**0.8486**	**0.8575**	**0.9357**
**Multi classification**					
ResNet18 (10)	0.7549	0.7976	0.8186	0.8080	0.9353
ResNet34 (10)	0.7712	0.8220	0.8485	0.8349	0.9589
DenseNet (32)	0.6454	0.6685	0.7289	0.6974	0.8235
SENet_18 (36)	0.5767	0.5940	0.6434	0.6177	0.7666
ECANet_18 (37)	0.6650	0.6895	0.7305	0.7094	0.8785
HRNet (39)	0.7320	0.7658	0.7940	0.7796	0.9226
GhostNet (35)	0.5898	0.6225	0.6913	0.6551	0.8184
RepVGG (30)	0.7173	0.7459	0.7717	0.7586	0.8872
Vision Transformer (11)	0.4771	0.4696	0.5421	0.5034	0.6256
CONVADD (15)	0.7216	0.5926	0.6268	0.6092	0.8339
Efficient B2 (41)	0.6862	0.7207	0.7869	0.7524	0.9114
**Our proposed**	**0.7924**	**0.8422**	**0.8596**	**0.8508**	**0.9635**

**Table 4:** Performance comparison of different models in DPC-Pre.

**Name**	**Accuracy (%)**	**Recall (%)**	**Precision (%)**	**F1-Score**	**AUC**
VGGNet (42)	48.0	50.1	49.5	0.498	0.539
ResNet18 (10)	90.4	93.2	93.2	0.933	0.987
ResNet34 (10)	92.4	96.3	95.4	0.959	0.995
ResNet50 (10)	87.4	90.1	90.1	0.901	0.963
ResNet101 (10)	85.9	88.5	88.8	0.887	0.945
DenseNet (32)	92.9	95.8	95.9	0.959	0.995
SENet_18 (36)	85.9	86.6	88.5	0.876	0.937
ShuffleNet (34)	89.9	92.6	92.8	0.927	0.976
MobileNet V2 (33)	93.9	96.8	96.8	0.969	0.995
ECANet_18 (37)	94.2	97.1	97.4	0.974	0.994
HRNet (39)	81.3	83.9	83.9	0.839	0.940
GhostNet (35)	88.4	91.2	91.1	0.911	0.973
RepVGG (30)	88.4	91.1	92.2	0.917	0.987
Vision Transformer (11)	82.5	86.5	80.9	0.836	0.864
CONVADD (15)	92.9	95.9	95.9	0.959	0.989
Efficient B2 (41)	87.3	90.1	90.1	0.901	0.968
Our Proposed	95.5	97.9	98.0	0.979	0.997

**Table 5:** Performance comparison of different models in DPC-SF.

**Name**	**Accuracy (%)**	**Recall (%)**	**Precision (%)**	**F1-Score**	**AUC**
VGGNet (42)	38.9	33.4	40.4	0.365	0.478
ResNet18 (10)	87.5	91.3	90.8	0.910	0.967
ResNet34 (10)	74.5	77.6	78.3	0.779	0.902
ResNet50 (10)	76.5	80.0	79.6	0.797	0.912
ResNet101 (10)	79.8	83.2	83.3	0.832	0.931
DenseNet (32)	87.5	90.9	91.0	0.909	0.971
SENet_18 (36)	78.5	81.4	82.2	0.817	0.912
ShuffleNet (34)	71.7	75.1	74.5	0.747	0.889
MobileNet V2 (33)	89.7	93.1	93.2	0.931	0.978
ECANet_18 (37)	86.9	90.3	90.5	0.903	0.967
HRNet (39)	86.8	90.0	90.2	0.901	0.968
GhostNet (35)	86.6	89.9	90.1	0.899	0.971
RepVGG (30)	89.8	81.6	94.8	0.877	0.973
Vision transformer (11)	83.5	79.7	77.5	0.785	0.806
CONVADD (15)	82.9	85.5	85.6	0.855	0.946
Efficient B2 (41)	87.3	91.1	90.6	0.908	0.990
Our proposed	93.7	97.3	97.3	0.973	0.998

In the KAC dataset (shown in Table 3), we conducted comprehensive evaluations of the proposed method's effectiveness by comparing its performance in binary classification and multi-classification tasks. In the binary classification task, our proposed method outperforms the other comparative models in all evaluation metrics, with an accuracy of 0.8431 and an F1-score of 0.8575. It significantly outperforms most deep learning models, such as the DenseNet and ResNet series, and exhibits a strong discriminative ability. In the multi-classification task, although the overall recognition difficulty is higher and the performance of the models decreases, our proposed method still achieves the optimal results. It achieves an accuracy of 0.7924 and an F1-score of 0.8508. Our method, as demonstrated by its recall and precision metrics of 0.8422 and 0.8596, respectively, accurately discriminates between multiple classes. On the DPC-Pre dataset (shown in Table 4), most of the deep networks can achieve high recognition accuracy on this dataset, among which MobileNetV2 (93.9%), ResNet34 (92.4%), and DenseNet (92.9%) perform well. In contrast, however, our proposed method achieves the best performance, with an accuracy of 95.5% and an F1-score of 0.979. On the DPC-SF dataset (see Table 5), the performance of most models degrades due to increased task complexity. However, our proposed method still maintains a significant advantage, outperforming the other compared methods in accuracy (93.7%) and F1-Score (0.973). It still offers substantial improvement in all metrics. In summary, the experimental results on the KAC, DPC-Pre, and DPC-SF datasets demonstrate that our proposed method consistently outperforms existing mainstream models and exhibits superior discriminative ability.

### 4.4 Ablation study

To further validate the effectiveness of each module in our proposed method, we conducted a series of ablation experiments on the KAC dataset. Specifically, we start from a baseline architecture and progressively extend the proposed modules. The compared models are listed in Table 6. The specific results are shown in Table 7. The basic single-branch structure exhibits limited performance, with accuracies of 0.7810 (Branch I) and 0.7843 (Branch II). Fusing the two into a dual branch results in a significant performance improvement. A slight decrease in performance was observed after adding enhancement modules to Dual Branch, probably due to the introduced structural complexity not working effectively. The further introduction of the attention mechanism improved several metrics, including an accuracy of 0.8319 and an AUC of up to 0.9368. Ultimately, the proposed method is optimal, achieving an accuracy of 0.8431 and an F1 score of 0.8575, which proves the effectiveness of the overall architectural design. In the multi-classification task, the single-branch model performed weakly, with a highest accuracy of 0.6764, while the dual-branch model significantly improved performance, achieving an accuracy of 0.7385. The attention mechanism was equally effective. The final proposed method also achieves the best results in the multi-classification task, with an accuracy of 0.7924 and an F1 score of 0.8508, further validating the adaptability and superiority of the proposed module in complex tasks. In summary, the ablation experiments fully demonstrate the effectiveness and complementarity of the overall design in the classification task, showing that the proposed method has robust generalization ability and high discriminative performance.

**Table 6:** The compared models of the ablation experiment.

**Model name**	**Conv_5**	**Conv_7**	**Cross-scale feature fusion**	**Attention**
Branch I - small	✓			
Branch II - large		✓		
Dual branch	✓	✓		
Dual branch enhance	✓	✓	✓	
Dual branch attention	✓	✓		✓
Our proposed	✓	✓	✓	✓

**Table 7:** Ablation study results on the binary classification task.

**Name**	**Accuracy**	**Recall**	**Precision**	**F1-Score**	**AUC**
**Binary classification**					
Branch I - small	0.7810	0.8034	0.7856	0.7944	0.9006
Branch II - large	0.7843	0.7986	0.7794	0.7889	0.8991
Dual branch	0.8310	0.8508	0.8290	0.8397	0.9288
Dual branch enhance	0.7672	0.7627	0.7430	0.7527	0.8752
Dual branch attention	0.8319	0.8566	0.8386	0.8475	0.9368
**Our proposed**	**0.8431**	**0.8667**	**0.8486**	**0.8575**	**0.9357**
**Multi classification**					
Branch I - small	0.6764	0.7130	0.7477	0.7299	0.8896
Branch II - large	0.6960	0.7366	0.7749	0.7553	0.9148
Dual branch	0.7385	0.7759	0.8026	0.7891	0.9314
Dual branch enhance	0.7156	0.7635	0.7925	0.7777	0.9264
Dual branch attention	0.7679	0.7586	0.7895	0.7737	0.9248
**Our proposed**	**0.7924**	**0.8422**	**0.8596**	**0.8508**	**0.9635**

### 4.5 Confusion matrix

**Figure 2:**
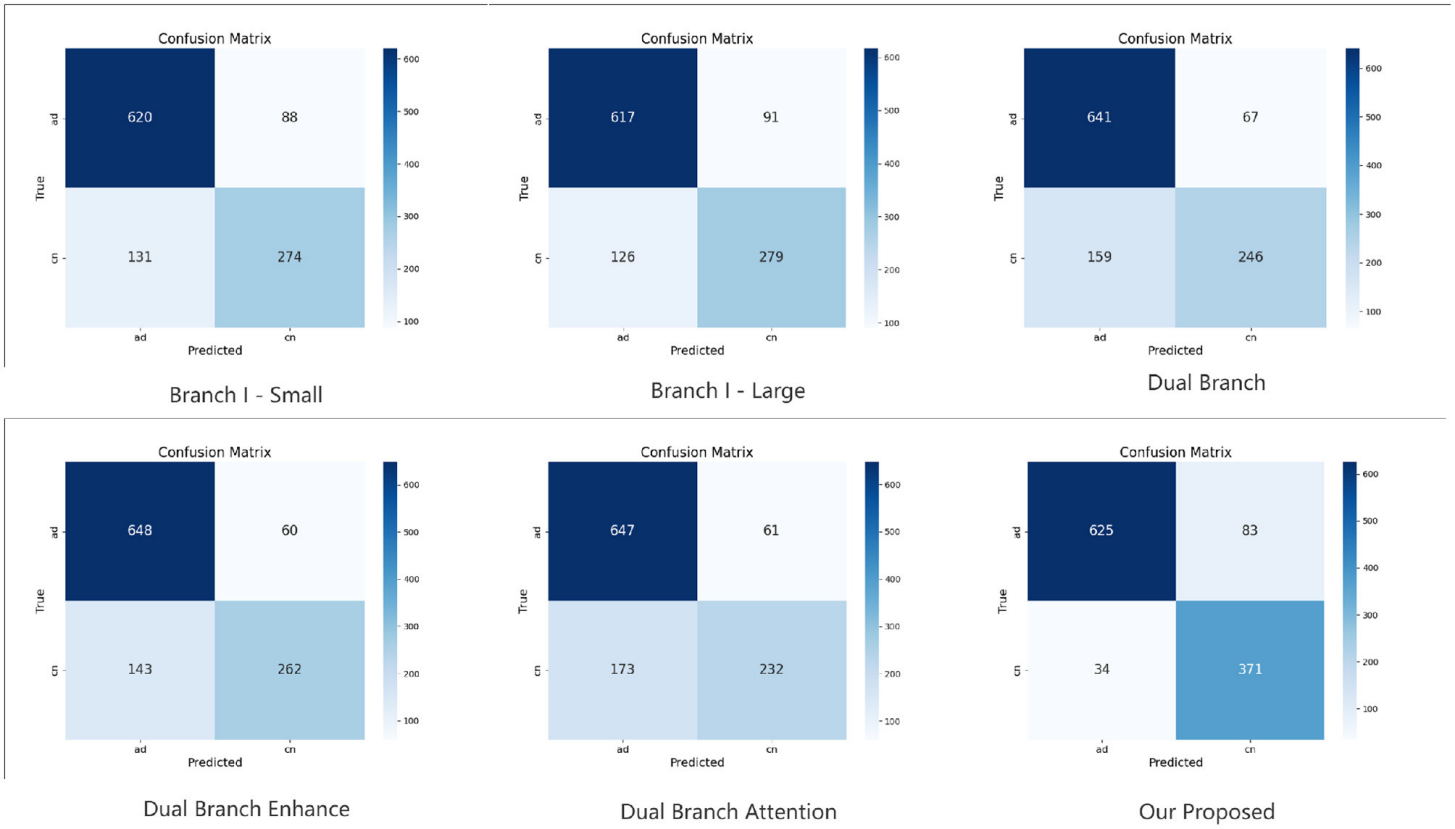
Confusion matrix results of binary classification task in KAC dataset.

**Figure 3:**
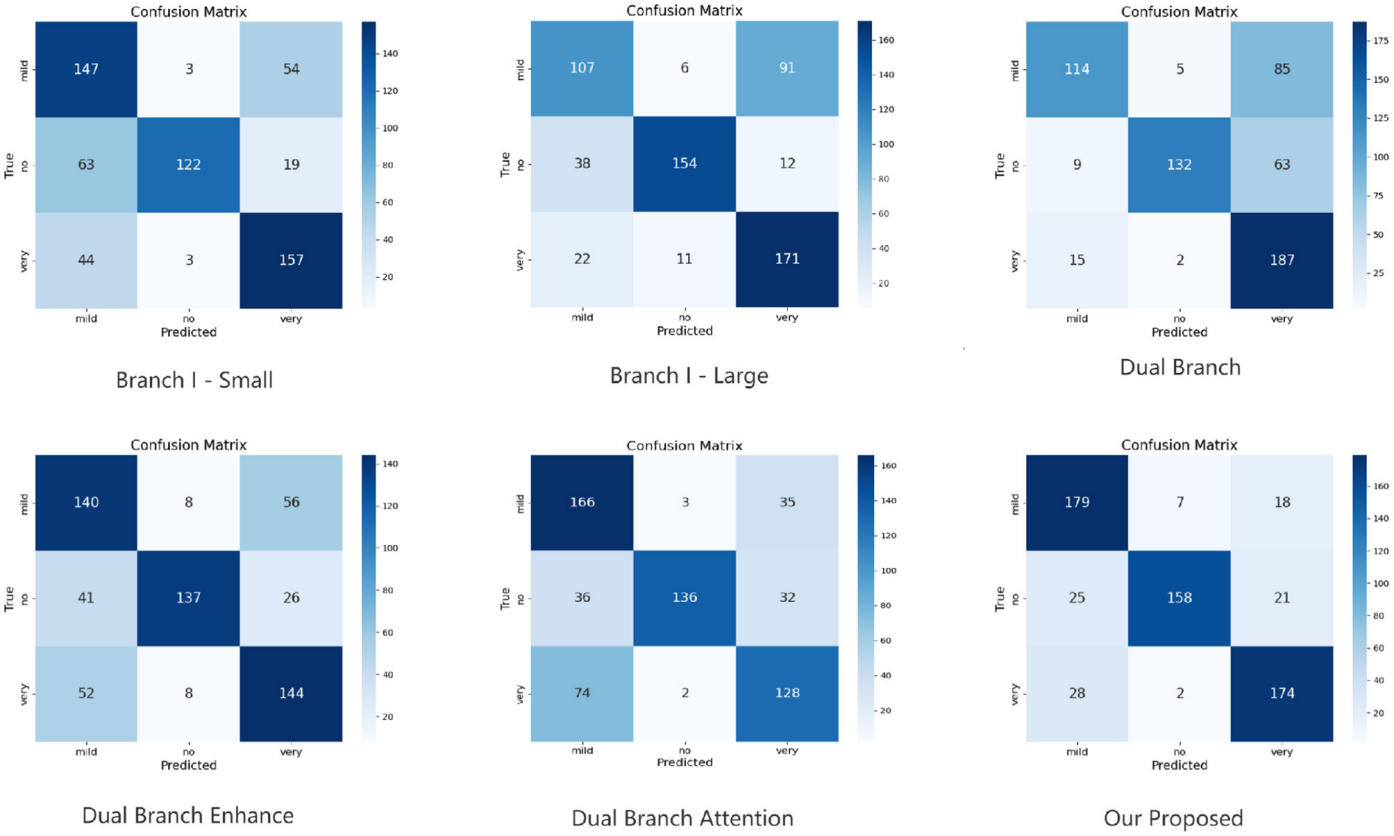
Confusion matrix results of multi-classification task in KAC dataset.

To further investigate the classification performance of each ablation model, we conduct and visualize its confusion matrix. The confusion matrix results of binary and multi-classification are shown in Figures 2, 3. The results demonstrate a significant improvement in classification accuracy as the model structure is gradually enhanced. The initial “Branch I - Small” model had high rates of false positives and false negatives in its predictions. The “Branch I - Large” model improved the prediction accuracy and reduced the false negatives. Then, the classification performance was further enhanced by introducing the “Dual Branch” structure. The model will better recognize Alzheimer's disease, although many false positives will still exist. Ultimately, the proposed model performs well in the classification task by combining all the improvement strategies. This shows that enhancing the model structure and introducing the attention mechanism effectively improve the classification accuracy and optimize the model's generalization ability.

### 4.6 Model interpretability

**Figure 4:**
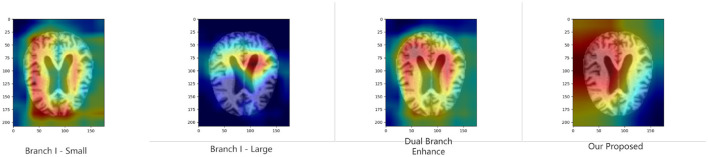
Grad-CAM results of binary classification task in KAC dataset.

**Figure 5:**
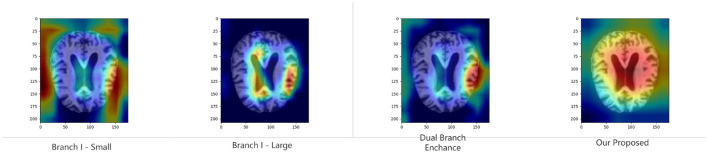
Grad-CAM results of multi-classification task in KAC dataset.

To further validate the proposed module's ability to improve model discrimination and interpretability, we performed Grad-CAM (40) analysis on the model under various ablation settings. Specifically, we compare the heat maps generated by the whole model with those generated by the model with the key module removed. As shown in Figures 4, 5, the region of attention of the whole model is more focused and highly aligned with the lesion region. In contrast, the model with the modules removed tends to focus on scattered or irrelevant areas. This result shows that the modules we introduced not only bring performance improvements in quantitative metrics but also prompt the model to focus more on medically significant regions in the decision-making process. It enhances the interpretability and reliability of the model in clinical diagnosis.

## 5 Discussion

In recent years, the application of deep learning in Alzheimer's disease (AD) image recognition has achieved remarkable results; however, existing methods still face numerous challenges in handling complex brain structural features. Specifically, multi-scale convolutional neural networks often struggle to achieve effective inter-scale interaction when fusing information from different spatial scales. This results in a limited representation of information in key focal regions. Meanwhile, the local receptive fields of traditional convolutional operations hinder the model's ability to effectively capture long-range dependencies between features at different scales. To this end, the X-FASNet proposed in this paper introduces three core strategies at the structural design level. First, the multi-scale feature extraction structure is employed to fully capture image information across different receptive fields, thereby enhancing the model's sensitivity to Alzheimer's disease. Second, the cross-scale feature-aware self-attention is designed to facilitate information interaction and integration between different scales. In the experimental analysis, we further verify the effectiveness of the above structural design through a series of quantitative evaluations and visualization methods. Furthermore, the CAM-based results indicate that the whole model can focus more on typical regions associated with Alzheimer's disease compared to the control model with key modules removed. This suggests that the introduced structural design not only improves the classification performance but also aligns the model's focus mechanism more closely with the actual clinical regions of concern, thereby enhancing the credibility of the model output.

The effectiveness of the proposed structural design is validated through a series of experiments. First, the ablation results (Table 7) show that our model consistently outperforms all baseline variants across multiple evaluation metrics, confirming that the three core strategies make a significant contribution to performance enhancement. Further, the multi-scale feature extraction provides richer information for diagnosis, while the cross-scale feature-aware self-attention strengthens the feature representation capability. In addition, these quantitative improvements are further supported by qualitative evidence from Grad-CAM visualizations (Figures 4, 5). Compared with models using only single-scale branches or lacking attention mechanisms, our proposed model demonstrates more focused, semantically meaningful, and structurally coherent attention regions. The activation patterns are concentrated in brain areas closely associated with AD pathology, indicating stronger clinical consistency and interpretability.

However, the method presented still has some limitations. First, due to the high cost of acquiring high-quality neuro-imaging data related to Alzheimer's disease, the size and diversity of training data are still limited at this stage. In addition, this study utilizes unimodal structural MRI data, which, to some extent, limits the model's ability to comprehensively represent the complex pathological mechanisms of AD. Second, this study was evaluated based on publicly available datasets. Its image acquisition conditions are relatively uniform, and it lacks heterogeneous data validation from different centers, different devices, or different populations. In the future, we will consider combining multi-modal data, such as PET images, to enhance the model's ability to express complex pathological mechanisms. At the same time, data from different hospitals or devices are introduced to construct cross-center validation sets, enhancing the robustness and generalization performance of the model in real-world scenarios. Beyond dataset expansion, we will also consider verifying the practical application effectiveness of the model. Firstly, we will collaborate with clinical institutions to conduct prospective studies and integrate the model into computer-aided diagnosis (CAD) systems for evaluation in practical workflows. Additionally, we aim to test the model's inference efficiency and deployment feasibility on hospital-grade hardware to ensure that it meets the performance requirements for clinical deployment. These efforts will help transition our approach from research validation to practical implementation.

## 6 Conclusion

In this study, an X-FASNet is proposed for the early diagnosis task of Alzheimer's disease and validated based on Alzheimer's disease MRI data. The model effectively captures both local and global features in brain images through cross-scale information fusion. Experiments on multiple publicly available datasets demonstrate that X-FASNet outperforms existing methods in terms of classification performance. Meanwhile, the visualization analysis, combined with the model interpretability, shows that the model can focus on clinically significant brain regions stably, providing intuitive support for the model's discrimination process.

## Data Availability

The original contributions presented in the study are included in the article/supplementary material, further inquiries can be directed to the corresponding author.

## Footnotes

^1^ https://www.kaggle.com/datasets/borhanitrash/alzheimer-mri-disease-classification-dataset

^2^ https://aistudio.baidu.com/datasetdetail/98269
